# T‐cell responses in COVID‐19 survivors 6−8 months after infection: A longitudinal cohort study in Pune

**DOI:** 10.1002/iid3.1238

**Published:** 2024-06-11

**Authors:** Poonam Suryawanshi, Bhagyashri Patil‐Takbhate, Prachi Athavale, Shahzad Mirza, Anuradha Tripathy, Shubhangi Kanitkar, Sachin Shivnitwar, Madhusudan S. Barthwal, Sachin Dole, Hanumant Chavan, Priyanka Jali, Sujata Pawale, Dhanashree Kakad, Arjun Lal Kakrani, Jitendra Bhawalkar, Madhura Gandhi, Sarika Chaturvedi, Mahesh Karandikar, Srikanth Tripathy

**Affiliations:** ^1^ Central Research Facility, Dr D. Y. Patil Medical College, Hospital and Research Centre Dr D. Y. Patil Vidyapeeth, (deemed to be University) Pimpri Pune India; ^2^ Department of Microbiology, Dr D. Y. Patil Medical College, Hospital and Research Centre Dr D. Y. Patil Vidyapeeth, (deemed to be University) Pimpri Pune India; ^3^ Hepatitis Group ICMR‐National Institute of Virology Pune India; ^4^ Department of General Medicine, Dr D. Y. Patil Medical College, Hospital and Research Centre Dr D. Y. Patil Vidyapeeth, (deemed to be University) Pimpri Pune India; ^5^ Department of Respiratory Medicine, Dr D. Y. Patil Medical College, Hospital and Research Centre Dr D. Y. Patil Vidyapeeth, Pimpri, (deemed to be University) Pune India; ^6^ Dr D. Y. Patil Medical College, Hospital and Research Centre Dr D. Y. Patil Vidyapeeth, (deemed to be University) Pimpri Pune India; ^7^ Department of Community Medicine, Dr D. Y. Patil Medical College, Hospital and Research Centre Dr D. Y. Patil Vidyapeeth, (deemed to be University) Pimpri Pune India; ^8^ Dr D.Y. Patil Vidyapeeth, (deemed to be University) Pimpri Pune India

**Keywords:** coronavirus, COVID‐19 survivors, flow cytometry, lymphocyte subsets, SARS‐CoV‐2

## Abstract

**Background:**

The severe acute respiratory syndrome coronavirus 2 (SARS‐CoV‐2) immune response is crucial for disease management, although diminishing immunity raises the possibility of reinfection.

**Methods:**

We examined the immunological response to SARS‐CoV‐2 in a cohort of convalescent COVID‐19 patients in matched samples collected at 1 and 6−8 months after infection. The peripheral blood mononuclear cells were isolated from enrolled study participants and flow cytometry analysis was done to assess the lymphocyte subsets of naive, effector, central memory, and effector memory CD4+ or CD8+ T cells in COVID‐19 patients at 1 and 6−8 months after infection. Immunophenotypic characterization of immune cell subsets was performed on individuals who were followed longitudinally for 1 month (*n* = 44) and 6−8 months (*n* = 25) after recovery from COVID infection.

**Results:**

We observed that CD4 +T cells in hospitalized SARS‐CoV‐2 patients tended to decrease, whereas CD8+ T cells steadily recovered after 1 month, while there was a sustained increase in the population of effector T cells and effector memory T cells. Furthermore, COVID‐19 patients showed persistently low B cells and a small increase in the NK cell population.

**Conclusion:**

Our findings show that T cell responses were maintained at 6−8 months after infection. This opens new pathways for further research into the long‐term effects in COVID‐19 immunopathogenesis.

## Graphical abstract

It is showing study participants, study methodology, and results.

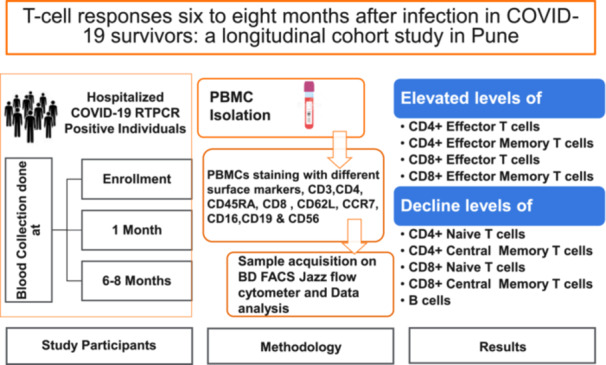

## 1 INTRODUCTION

The severe acute respiratory syndrome coronavirus 2 (SARS‐CoV‐2) infection that led to the coronavirus disease in 2019 (COVID‐19) is still a hazard to the general public's health. Since the beginning of the pandemic, there have been more than 5.3 million recorded deaths and over 275 million confirmed cases (WHO weekly epidemiological update on COVID‐19, December 21, 2021). The predominant symptom of COVID‐19 is a respiratory illness with symptoms ranging from asymptomatic or moderate infection to severe symptoms necessitating intensive care unit (ICU) hospitalization.^1^

To control and eradicate viral infections, a unique adaptive immune response must be developed. More specifically, virus‐specific T and B cells are stimulated, grow, and eventually develop into effector cells in response to infection. Neutralizing antibodies and memory B and T cells, which are specific to the viral antigen survive long after the infection has been eradicated. This memory immune response, which is activated during vaccination, is crucial in the prevention of reinfection. To comprehend the emergence and persistence of such protective immunity, it is crucial to characterize in detail the extent of specific adaptive immune responses in convalescent COVID‐19 patients with varying degrees of severity. For foreseeing and controlling potential future waves of infections in the general population, a deeper understanding of the mechanisms driving the development of protective immunological memory in recovered individuals is of paramount importance for public health.

The early response to SARS‐CoV‐2 infection in severely ill COVID‐19 patients is marked by significant immunological dysfunctions linked to a systemic inflammatory response and the emergence of altered innate and adaptive immune responses.^1^, ^2^ More particular, T cell response is significantly altered in critically ill COVID‐19 patients, and the most severe COVID‐19 patients have been reported as having severe lymphopenia, phenotypic, and functional T cell alterations.^3^ Therefore, it is still uncertain whether these critically ill patients can develop a strong and long‐lasting SARS‐CoV‐2 specific T cell response despite the presence of significant immunological changes during the stay in the hospital.

Considering this, the objective of the current investigation was to monitor the immunological response, including memory T cells specific to SARS‐CoV‐2, in samples obtained 1 and 6–8 months after infection from a cohort of convalescent critically ill COVID‐19 patients.

## 2 METHODOLOGY

### 2.1 Study design

The present study was approved by the Institutional Ethics Committee of Dr D. Y. Patil Vidyapeeth (Ref No‐DYPV/EC/634/2021 dated February 25, 2021) and carried out between March 2021 to February 2022 at Dr. D. Y. Patil Medical College, Hospital and Research Centre, Pune. The participants were ≥18‐years‐old male and female subjects who provided written informed consent before enrollment.

COVID‐19 patients were identified through a combination of epidemiological history, clinical symptoms, and whose nasal/throat swab positive test result by SARS‐CoV‐2 reverse transcription‐polymerase chain reaction (RT‐PCR) as per the WHO Interim Guidance.^4^ Patients with COVID‐19 were included in the study upon hospitalization. The initial blood samples for immunological investigations were collected around the 5th day of COVID‐19 infection, with an average deviation of ±2 days, and two subsequent samples were collected at 1 and 6−8 months, respectively, measured from the time of enrollment in the study. Twenty‐one healthy individuals were recruited as uninfected controls for immunophenotype comparison. All uninfected controls had no history of SARS‐CoV‐2 infection during the previous 6 months and were confirmed negative by COVID‐19 RT PCR test. The study was carried out in compliance with good clinical practices, including the International Conference on Harmonization Guidelines and the Declaration of Helsinki.

### 2.2 Patient characteristics

The COVID‐19 patients were administered an approved questionnaire upon enrollment to collect information regarding COVID‐19‐related symptoms before hospitalization and smoking behaviors. The date of COVID‐19 diagnosis, highest level of care received, the maximum amount of oxygen supplementation required (unit, oxygen need 5 L/min supplemented by High Flow Nasal Oxygen or Continuous Positive Airway Pressure, and the critical care provided (ICU, with or without mechanical ventilation) for COVID‐19 illness were all gathered from digital medical records. The patients with severe/moderate illness were those who required clinical care and required oxygen support. In brief, the moderate patients had oxygen saturation (SpO_2_) of 90%−94%, mostly required supplement oxygen of 2−6 L and did not require noninvasive ventilation. The severe patients had oxygen saturation (SpO_2_) of ≤90%, mostly required supplement oxygen of 4−20 L and noninvasive ventilation or high‐flow oxygen.

### 2.3 Peripheral blood mononuclear cells (PBMC) isolation

EDTA anticoagulated peripheral blood (5 mL) was collected from each individual. All samples were tested within 6 h of blood collection. The PBMCs and plasma were separated by density gradient centrifugation according to standard protocols and the plasma was stored at −80°C till further use.

### 2.4 Flow cytometry for immunophenotyping

For analysis of surface markers, fresh PBMCs were incubated for 30 min at room temperature in Stain Buffer (BD Biosciences) with optimal concentrations of the fluorochrome conjugated antibodies. Lymphocyte subsets were determined using the monoclonal antibodies as follows: CD3 APCH7 (Clone—SK‐7), CD4 BV480 (Clone—SK‐3), CD8 FITC (Clone—RPA‐T8), CD62L APC (Clone—Dreg 56), CCR7 PE (Clone—2‐L1‐A), CD45RA BV421 (Clone—5H9), CD56 BV480 (Clone—NCAM 16.2), and CD16 FITC (Clone—3G8) (BD Biosciences). The cells were further washed with stain buffer, fixed with 1% formaldehyde in PBS and acquired to obtain 100,000 gated lymphocyte events on FACS Jazz (BD Biosciences). Gating strategy for identification of different immune cells and subsets was depicted in Supporting Information Material 1. The data was analyzed using FACS Flow Jo software V10.7 (BD Biosciences).

### 2.5 Detection of anti‐SARS‐CoV‐2 antibodies

Plasma samples were assessed for the presence of anti‐SARS‐CoV‐2‐specific binding antibodies using the commercially available Abbott SARS‐CoV‐2 IgG assay (M/s Abbott Laboratories' Diagnostics Division) and conducted on the ARCHITECT machine as per the manufacturer's instructions. Test results were quantified as anti‐SARS‐CoV‐2 index values, where an index >1.4 was interpreted as a positive result.

### 2.6 Statistical analysis

SPSS (Statistical Package for Social Sciences) version 26.0; IBM was used to analyze the data. The Shapiro–Wilk test was used to assess the distribution of the data set. Total counts (frequency), percentages, means, and standard deviations were generated as part of descriptive statistics for patient demographics. For continuous variables, an independent sample *t*‐test/Mann–Whitney *U* test was employed, and for categorical connections, a *χ* ^2^ or Fisher exact test was utilized. Spearman's rank correlation was used for the correlation analysis, and a *p* value of ≤.05 was considered statistically significant.

## 3 RESULTS

### 3.1 Clinical characteristics of COVID‐19 patients

During an 18‐month period (March 2021 to September 2022), blood samples were collected from hospitalized COVID‐19 patients. A subset of donors was followed longitudinally at 1 month (*n* = 44) and up to 6–8 months (*n* = 25). Our analysis focused on the effect of COVID‐19 on various T cell subsets and B cell immune responses. The clinical and demographic characteristics of the hospitalized COVID‐19 cohort are summarized in Table 1. Our hospitalized COVID‐19 cohort represents the infection in wider society in terms of biological sex distribution, encompassing 25 males and 19 females, and a mean age of 51.4 years (Table 1). Out of the total participants, only 12 patients took their COVID‐19 vaccination during the study period, while the remaining individuals did not receive the vaccine. Table 2 provides the biochemical laboratory parameters for hospitalized COVID‐19 patients at enrollment in the study. The disease categorization for the patients were moderate or severe as per the guidelines given by the National Institutes of Health.^5^

**Table 1:** Demographic and clinical characteristics of the enrolled study participants.

	Healthy controls (21)	COVID‐19 patients (*n* = 44)
Age (years) mean ± SD	41.7 ± 14.5	51.4 ± 13.2
Sex		
Male *n* (%)	18 (85.7)	25 (56.81%)
Female *n* (%)	3 (14.3)	19 (43.19%)
Height (cm)	167.7 (2.8)	158.5 ± 13.4
Weight (kg)	67.2 (8.0)	67.9 ± 13.3
BMI (kg/m)^2^	23.98 (2.7)	27.1 ± 5.4
Comorbidity *n* (%)		
Diabetes mellitus	NIL	12 (27.9%)
Hypertension	NIL	16 (37.2%)
Liver failure	NIL	1 (2.3%)
Kidney disease	NIL	3 (7%)
Cancer	NIL	0
COPD	NIL	0
Signs and symptoms at baseline *n* (%)		
Rigors (shaking chills)	NA	10 (23.3%)
Diarrhea (≥3 stools/24 h)	NA	8 (18.6%)
Cough	NA	30 (69.8%)
Breathing difficulty	NA	36 (83.7%)
Dysuria	NA	3 (7%)
Abdominal pain	NA	2 (4.7%)
Myalgia	NA	27 (62.8%)
Arthralgia	NA	3 (7%)
Sore throat	NA	10 (23.3%)
Anosmia	NA	4 (9.3%)
Nausea/vomiting	NA	6 (14%)
Altered mental status	NA	0
Headache	NA	9 (20.9%)
Rash	NA	1 (2.3%)
Loss of smell and/or taste	NA	11 (25.6%)

Abbreviation: NA, not applicable.

**Table 2:** Biochemical laboratory findings for the enrolled study participants at baseline.

Biochemical test	Healthy controls (*n* = 21)	COVID‐19 patients (*n* = 44)	Reference range
HB (g/dL)	14.4 ± 1.4	12.4 ± 2.0	11.6−15.0
Total leukocyte count (cells/µL)	7357 ± 1055	6885 ± 3263	4000−10,000
Neutrophils (%)	55.8 ± 6.39	74.9 ± 11.9	40−80
lymphocytes (%)	33.4 ± 4.12	18.2 ± 10.7	20−40
Monocytes (%)	6.7 ± 1.7	5.9 ± 3.1	2−10
Eosinophils (%)	2.4 ± 1.5	0.8 ± 1.1	1−6
Platelets (cells/µL)	257,000 ± 58,672	210,609 ± 85,071	150,000−410,000
Creatinine (mg/dL)	0.83 ± 0.12	1.4 ± 1.7	0.6−1.35
Total bilirubin (mg/dL)	0.85 ± 0.47	0.5 ± 0.3	0.22−1.20
SGOT (U/L)	19.78 ± 5.5	124.0 ± 258.9	8−43
SGPT (U/L)	22 ± 12.4	87.0 ± 220.6	7−45
CRP (mg/dL)	NA	62.1 ± 47.8	<10
ESR (mm/h)	8.3 ± 3.1	48.7 ± 22.2	0−20
Sr. Ferritin	NA	599 ± 525	4.63−274.66
D‐Dimer (ng/mL)	NA	851 ± 862	0−500

Values given as mean ± SD.

### 3.2 Hospitalized SARS‐CoV‐2 patients had a persistent decline in CD4+ T cells, whereas CD8+ T cells gradually recover after 1 month

To assess the longitudinal effect of COVID‐19 on T cell populations, we sampled PBMCs from patients with COVID‐19 over a period of 6−8 months. PBMCs were analyzed by flow cytometry to investigate immunophenotype across the various T cell compartments.

**Figure 1:**
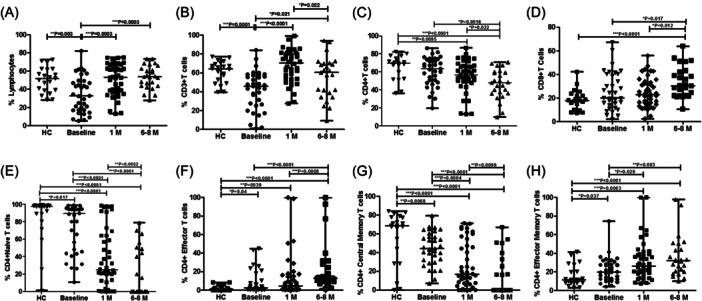
Flow cytometry analysis of T cells subsets. PBMCs were stained and acquired on flow cytometer. The bar graphs represent the comparison of percentages of immune cells and their subpopulation in healthy controls and COVID‐19 patients at different time point (A) lymphocytes, (B) CD3+ T cell, (C) CD4+ T cells, (D) CD8+ T cells, (E) CD4+ naïve T cells, (F) CD4+ effectors T cells, (G) CD4+ central memory T cells, and (H) CD4+ effector memory T cells profile. Data are represented as median with range. *p < .05, **p < .001, and ***p < .0001 significant. HC, healthy controls; PBMC, peripheral blood mononuclear cells.

At baseline, lymphocyte counts were notably lower (*p* = .03) compared to those of healthy controls. However, the lymphocyte populations recovered, with a substantial increase in cell counts at 1 month (*p* = .0003) that remained consistent until 6−8 month (*p* = .0003) reaching levels comparable to those of healthy controls (Figure 1A). Similarly, total T cells (CD3+ T cells) were significantly diminished at baseline (*p* = .0001) compared to healthy controls. Total T cells (CD3+ T cells) increased significantly after 1 month (*p* < .0001) and then dropped slightly at 6−8 months (*p* = .022). Nonetheless, T cell counts remained significantly higher at 1 and at 6−8 months than baseline (*p* = .021) and were comparable to healthy controls (Figure 1B) (Table 3).

**Table 3:** Percentages of different immune cell subsets in COVID‐19 patients at different time points.

	Healthy controls (*n* = 21)	Baseline (*n* = 44)	1 Month (*n* = 44)	6−8 Month (*n* = 25)
Lymphocytes	51.8 (28.40−72.6)	32.8 (7.06−82.1)	53.1 (13−75)	53.7 (27.8−73.4)
CD3+ T cells	64.4 (39.4−77.1)	45.8 (0.92−84.1)	70.55 (27.5−86.6)	60.5 (9.22−94.2)
CD4+ T cells	69.5 (36.2−82.2)	63.4 (19.5−86.2)	55.6 (0.00−86.6)	47.9 (9.69−70.7)
CD4 + naïve T cells (CD4 + CD45RA + CCR7 + CD62L+)	97.5 (1.09−99.4)	89.6 (10.5−98.9)	24.9 (0.0−98.5)	0 (0−79)
CD4 + effectors T cells (CD4 + CD45RA + CCR7‐CD62L−)	1.15 (0.04−8.39)	2.41 (0.2−44.9)	4.43 (0.0−99.9)	12.7 (1.37−99.7)
CD4 + central memory T cells (CD4 + CD45RA‐CCR7 + CD62L+)	68.5 (0.0−84.2)	44.3 (7.14−79.4)	16.65 (0.0−71.4)	0 (0−67)
CD4 + effector memory T cells (CD4 + CD45RA‐CCR7‐CD62L−)	11.5 (2.62−41.4)	19.9 (4.03−74.3)	25.95 (7.39−100)	32.2 (9.94−97.7)
CD8+ T cells	18 (0.0−42.1)	20 (1.96−67.2)	22.9 (0.0−55.9)	30.4 (10.4−63.9)
CD8 + naïve T cells (CD8 + CD45RA + CCR7 + CD62L+)	65.9 (0.37−89.9)	30.2 (0.19−92.3)	5.085 (0.0−80.5)	0.00642 (0−34.4)
CD8 + effectors T cells (CD8 + CD45RA + CCR7‐CD62L−)	18.3 (2.17−92.6)	39.4 (3.2−96.2)	54.1 (0.0−99.6)	58.9 (21.7−98.1)
CD8 + central memory T cells (CD8 + CD45RA‐CCR7 + CD62L+)	12.7 (0.98−33.3)	11.6 (0.11−32)	3.595 (0.0−29.9)	0.055 (0−22)
CD8 + effector memory T cells (CD8 + CD45RA‐CCR7‐CD62L−)	69.2 (4.76−93.6)	56.9 (3.55−93.2)	72.65 (0−99.7)	70.6 (47−99.4)
B Cells (CD3‐CD19+)	15.55 (5.4−34.1)	40.4 (0.11−77.9)	26.3 (0.29−68.5)	10.7 (2.3−63)
NK regulatory cells (CD3‐CD16‐CD56+)	2.91 (1.12−28.3)	1.84 (0.15−47.7)	3.96 (1.07−49.3)	4.28 (1.11−18.8)
NK cytotoxic cells (CD3‐CD16 + CD56+)	17.8 (3.58−41.6)	7.98 (0.47−35.7)	12.25 (0.019−42.9)	10.3 (0.16−52.5)
NKT cells (CD3 + CD56+)	8.6 (2.36−25.7)	9.52 (0.49−30.7)	8.85 (0.28−97.7)	8.37 (0.36−40.7)

All values are given in median with range.

When comparing CD4+ T cells, the percentages at baseline were comparable to healthy controls, however, a constant and significant reduction was observed at 1 month (*p* = .022) which is continued until 6−8 months (*p* = .0016) as compared to the healthy controls and baseline (Figure 1C). In relation to CD8+ T cells, the increase was significantly higher at 6−8 months compared to healthy controls (*p* < .0001), at baseline (*p* = .017), and at 1 month (*p* = .012) (Figure 1D) (Table 3).

Together, these data are reflective of the alterations in the levels of various immune cell types during COVID‐19, in line with observations made by others.^6^, ^7^, ^8^, ^9^, ^10^

### 3.3 COVID‐19 patients have a sustained, elevated effectors T cells and effector memory T cells population

The environment established by an infection, both locally and systemically, can cause changes in general memory T cell populations.^11^ We looked at how COVID‐19 affected naive, effector, and memory CD4+ and CD8+ T cells. Over time, distinct variations in the CD4+ and CD8+ T populations of COVID‐19 patients were detected (Table 3). The fraction of CD4+ naive T cells and CD4+ central memory cells decreased significantly with time when compared to healthy controls and at baseline (Figure 1E,G). At 6−8 months after discharge from hospital, there was a considerable increase in the CD4+ effectors and CD4+ effector memory T cell fractions compared to results obtained at the time of hospitalization and healthy controls (Figure 1F,H).

**Figure 2:**
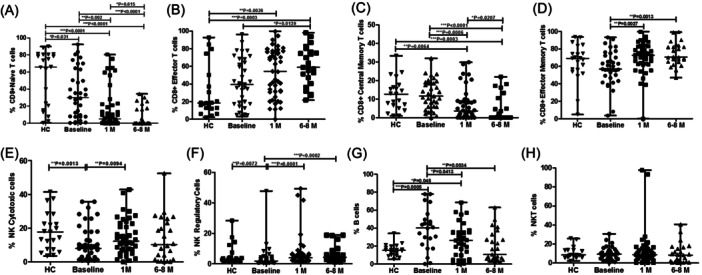
Flow cytometry analysis of CD8+ T cells, NK, and B cell subsets. The bar graphs represent the comparison of percentages of immune cells and their subpopulation in healthy controls and COVID‐19 patients at different time points (A) CD8+ naïve T cells, (B) CD8+ effectors T cells, (C) CD8+ central memory T cells, (D) CD8+ effector memory T cells, (E) NK cytotoxic cells, (F) NK regulatory cells, (G) B cells, and (H) NKT cells profile. Data are represented as median with range. *p < .05, **p < .001, and ***p < .0001 significant.

We observed that CD8+ T cells showed more sustained decrease in their naive and central memory as compared to healthy controls and from baseline to 6−8 months after enrollment (Figure 2A,C). The frequencies of CD8+ effectors and CD8+ T effector memory, on the other hand, increased from baseline to 6−8 months (Figure 2B,D) and also in comparison to healthy controls. Overall, we found alterations in the memory T cell pool, most notably in the CD4+ and CD8+ effector memory subsets, where the changes persisted until the end of the study.

### 3.4 Patients with COVID‐19 had a persistently low B cells and minimal increase in NK cell population

We further assessed B cells, NK cells, and NKT cells in peripheral blood to better understand lymphocyte attrition. The cytotoxic and regulatory fractions of NK cells increased slightly but not significantly with time compared to baseline and healthy controls (Figure 2E,F). There was a substantial decline in B cells from 1 month (*p* = .043) to 6−8 months (*p* = .0034) when compared to hospitalization and healthy controls (Figure 2G). Despite this, no significant changes in NKT cells were observed over time (Figure 2H).

### 3.5 Comparison of immune cell subsets between patients with moderate and severe conditions

**Figure 3:**
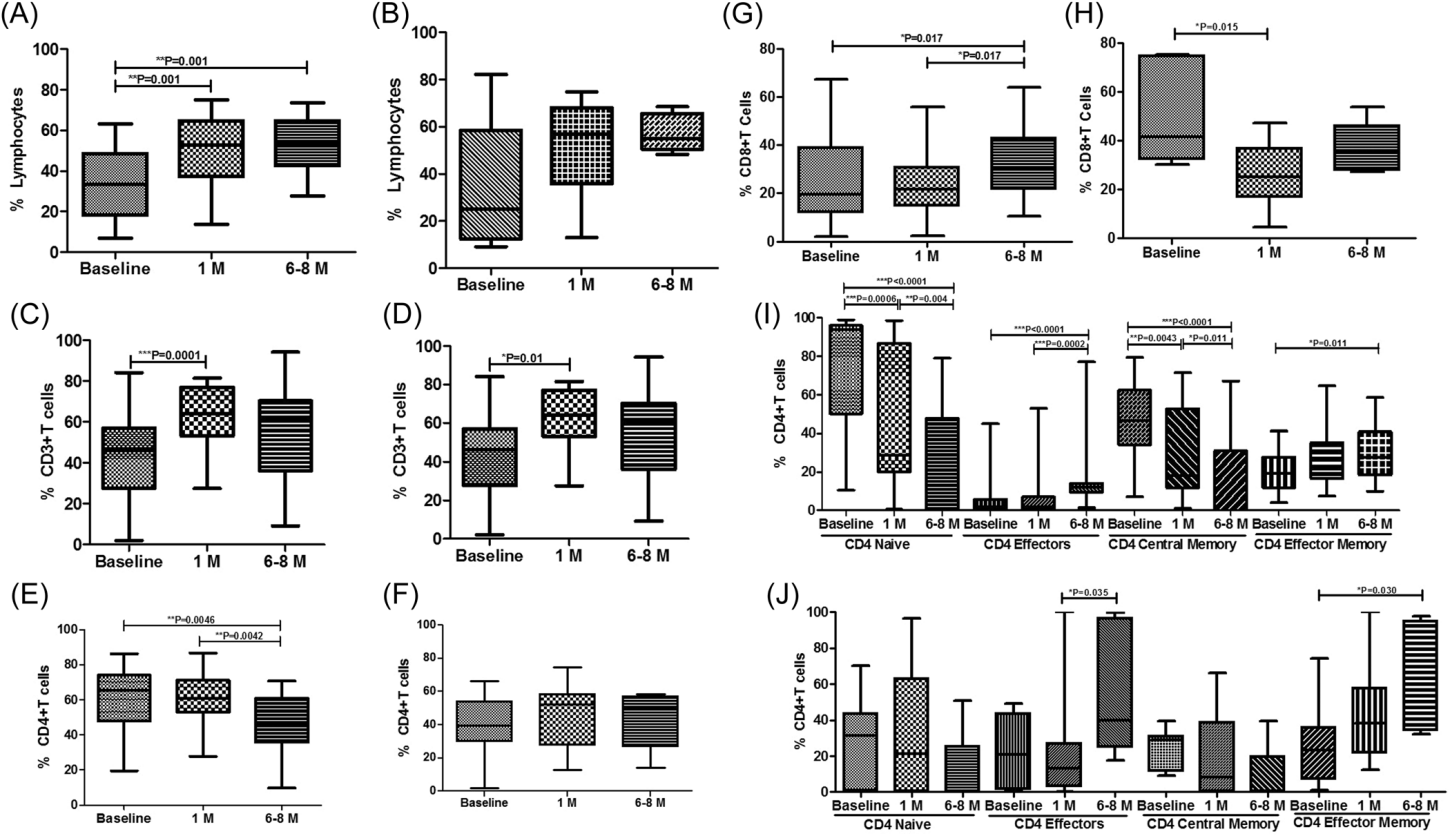
Flow cytometry analysis oimmune cell subsets in moderate and severe COVID‐19 cases. The bar graphs represent the comparison of percentages of immune cells and their subpopulation at different time point in moderate and severe COVID‐19 cases, respectively (A, B) lymphocytes, (C, D) CD3+ T cells, (E, F) CD4+ T cells, (G, H) CD8+ T cells, and (I, J) CD4+ memory T cells profile. Data are represented as median with range. *p < .05, **p < .001, and ***p < .0001 significant.

We further analyzed the differences in immune cell subsets in moderately and severely ill patients. We observed that lymphocytes and CD3+ T cells were increase over a period in both moderate and severely ill patients (Figure 3A–D). In case of CD4+ T cells, the percentages decreased significantly over a time while remained comparable in severe cases (Figure 3E,F). When comparing CD8+ T cells, the percentages were declined at 1 month and then recovered over a period of 6−8 months in both the cases (Figure 3G,H). The CD4+ T cell memory populations, CD4+ naïve T cells and CD4+ central memory cells decreased over a period of time while CD4+ effectors and CD4+ effector memory T cells increased over a span of 6−8 months from baseline in both moderate and severe cases (Figure 3I,J).

**Figure 4:**
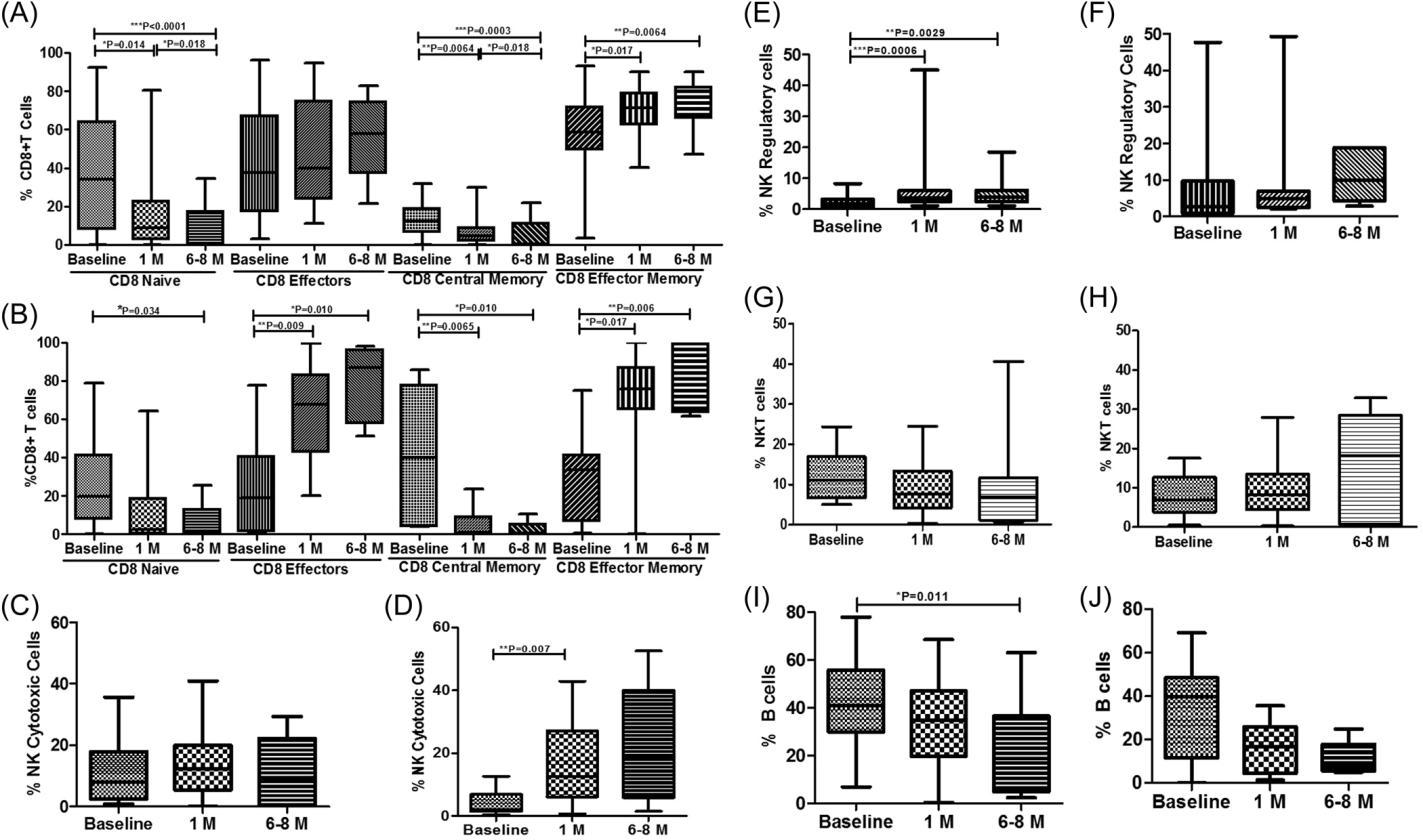
Flow cytometry analysis of CD8 T cells, B cells, and NK cells subsets in moderate and severe COVID‐19 cases. The bar graphs represent the comparison of percentages of immune cells and their subpopulation at different time point in moderate and severe COVID‐19 cases, respectively (A, B) CD8 + T cell memory subsets, (C, D) NK cytotoxic cells, (E, F) NK regulatory cells, (G, H) NKT cells, and (I, J) B cells. Data are represented as median with range. *p < .05, **p < .001, and *** *p* < .0001 significant.

In CD8+ T cell memory populations, CD8+ naïve T cells and CD8+ central memory cells decreased over a period of time while CD8+ effectors and CD8+ effector memory T cells increased over a span of 6−8 months from baseline in both moderate and severe cases (Figure 4A,B).

We further assessed B cells, NK cells, and NKT cells in moderated and severe cases. The cytotoxic (Figure 4C,D) and regulatory fractions (Figure 4E,F) of NK cells increased slightly but not significantly with time compared to baseline in both moderate and severe cases. However, no significant changes in NKT cells were observed over time in both cases (Figure 4G,H). When compared to baseline, there was a decrease in B cells from 1 to 6−8 months in both cases (Figure 4I,J) (Table 4).

**Table 4:** Comparison of percentages of different immune cell subsets in moderate and severe cases.

	Moderate			Severe		
	Baseline	1 Month follow‐up	6 Month follow‐up	Baseline	1 Month follow‐up	6 Month follow‐up
Lymphocytes	33.6 (7.06−63.2)	52.65 (13.8−75	53.55 (27.8−73.4)	25.2 (9.13−82.1)	56.95 (13−74.7)	55.1 (48.3−68.5)
CD3 + T cells	46.4 (2.04−84.1)	64 (27.5−81.5)	61.65 (9.22−94.2)	49.9 (0.92−74.5)	79.75 (29.2−99.2)	57.5 (29.2−92.9)
CD4 + T cells	65.2 (19.5−86.2)	60.9 (27.8−86.6)	46.3 (9.69−70.7)	39.7 (1.91−66.4)	52.2 (13−74.5)	50.1 (14.4−58.6)
CD4 + naïve T cells (CD4 + CD45RA + CCR7 + CD62L+)	93.75 (10.5−98.9)	28.8 (0.59−98.5)	0 (0−79)	31.5 (0.22−70.1)	21.2 (0−96.5)	0.00642 (0−50.6)
CD4 + effectors T cells (CD4 + CD45RA + CCR7‐CD62L−)	1.945 (0.2−44.9)	1.91 (0.25−52.9)	12.25 (1.37−77)	21 (0.94−49.2)	13 (0−99.9)	39.9 (17.7−99.7)
CD4 + central memory T cells (CD4 + CD45RA‐CCR7 + CD62L+)	46.35 (7.14−79.4)	18.05 (1.03−71.4)	0.001185 (0−67)	28.8 (9.1−39.3)	8.44 (0−66)	0 (0−39.6)
CD4 + effector memory T cells (CD4 + CD45RA‐CCR7‐CD62L−)	19.45 (4.03−41.2)	21.5 (7.39−64.6)	27.55 (9.94−58.6)	23.1 (1.21−74.3)	38.35 (12.4−100)	44 (32.2−97.7)
CD8 + T cells	19.55 (1.96−67.2)	21.65 (2.39−55.9)	30.2 (10.4−63.9)	41.5 (30.1−75.4)	25.25 (4.47−47.1)	35.8 (27.1−53.7)
CD8 + naïve T cells (CD8 + CD45RA + CCR7 + CD62L+)	34.25 (0.19−92.3)	8.69 (0.23−80.5)	0.00321 (0−34.4)	19.7 (0.32−78.8)	2.535 (0−64.2)	0.00938 (0−25.6)
CD8 + effectors T cells (CD8 + CD45RA + CCR7‐CD62L−)	37.65 (3.2−96.2)	39.95 (11.3−94.5)	57.9 (21.7−82.8)	19 (1.28−77.5)	66.5 (0−99.6)	86.9 (51.4−98.1)
CD8 + central memory T cells (CD8 + CD45RA‐CCR7 + CD62L + )	12.25 (0.11−32)	4.67 (0.044−29.9)	0.236 (0−22)	40 (3.85−85.7)	1.02 (0−23.7)	0.055 (0.016−10.5)
CD8 + effector memory T cells (CD8 + CD45RA‐CCR7‐CD62L−)	58.5 (3.55−93.2)	71.3 (40.2−90)	70.65 (47−90.2)	33.7 (0.73−75)	75.9 (0−99.7)	68.3 (61.7−99.4)
B cells (CD3‐CD19+)	40.9 (6.85−77.9)	34.8 (0.29−68.5)	16.85 (2.3−63)	39.9 (0.11−69.2)	16.55 (1.28−35.5)	8.66 (4.9−24.9)
NK regulatory cells (CD3‐CD16‐CD56+)	1.755 (0.41−8.26)	3.715 (1.07−45)	3.8 (1.11−18.5)	2.67 (0.15−47.7)	4.885 (2.14−49.3)	9.99 (2.84−18.8)
NK cytotoxic cells (CD3‐CD16 + CD56+)	8.115 (0.78−35.7)	12.25 (0.019−40.9)	8.845 (0.16−29.3)	2.03 (0.47−12.7)	12.5 (0.79−42.9)	18.8 (1.52−52.5)
NKT cells (CD3 + CD56+)	11.1 (5.02−24.4)	7.67 (0.34−24.5)	6.915 (0.73−40.7)	6.955 (0.49−17.5)	9.725 (0.28−97.7)	18.1 (0.36−32.9)

All values are given in median with range.

### 3.6 Paired analysis of immune subsets in COVID‐19 patients at baseline and 6 months

**Figure 5:**
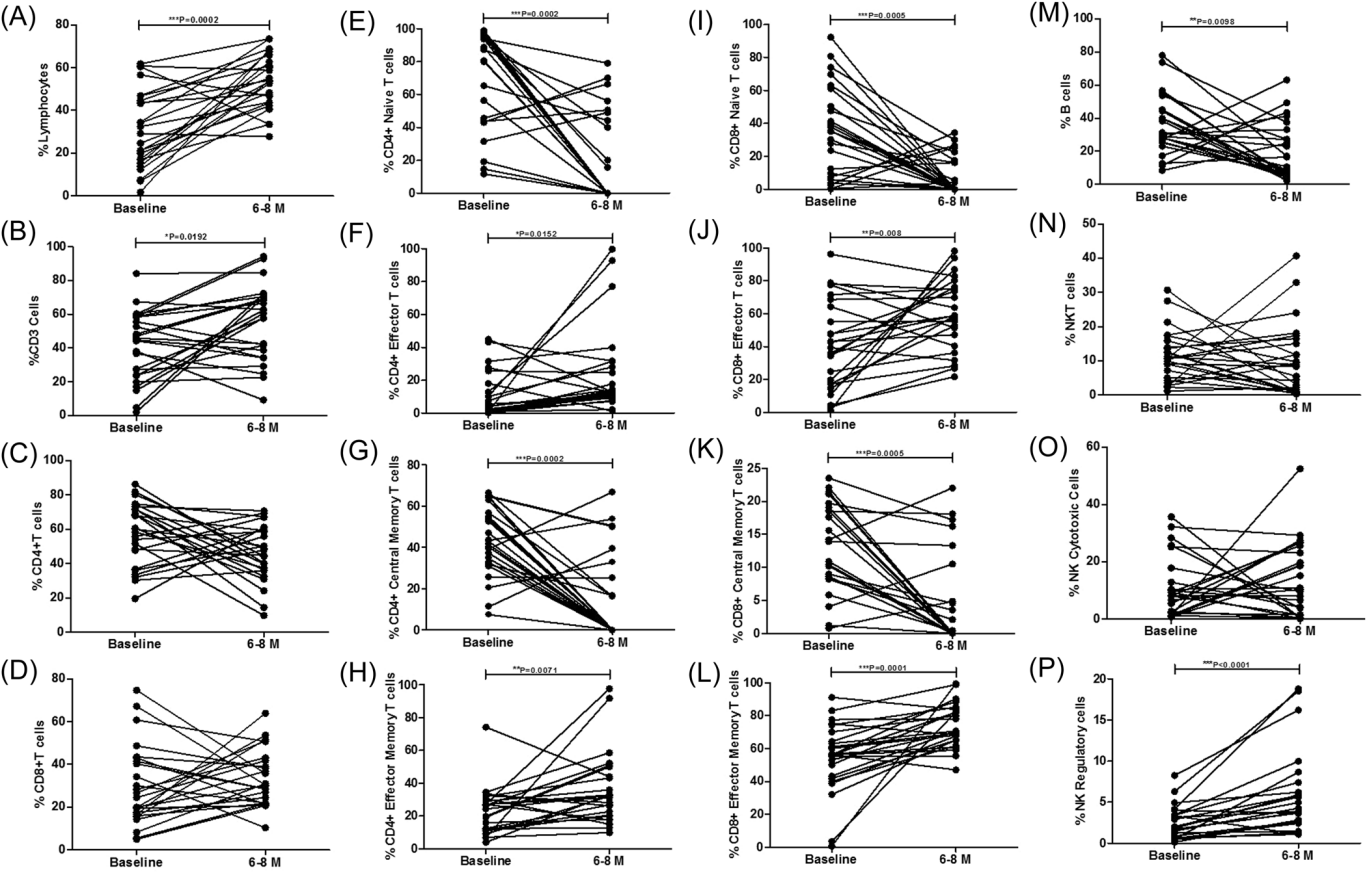
Paired analysis of different immune cell subsets in 25 COVID‐19 patients at baseline and after 6−8 months. The paired line graph shows percentages of different immune cell subsets in 25 COVID‐19 patients at baseline and after 6−8 months as follows (A) lymphocytes, (B) CD3 + T cell, (C) CD4 + T cells, (D) CD8 + T cells, (E) CD4+ naïve T cells, (F) CD4+ effectors T cells, (G) CD4+ central memory T cells, (H) CD4+ effector memory T cells, (I) CD8+ naïve T cells, (J) CD8+ effectors T cells, (K) CD8+ central memory T cells, (L) CD4+ effector memory T cells, (M) B cells, (N) NKT cells, (O) NK cytotoxic cells, and (P) NK regulatory cells profile. *p < .05, **p < .001, and ***p < .0001 significant.

Further we have analyzed 25 COVID‐19 patients paired data to address any bias due to follow‐up. We have found that percentages of lymphocytes, CD3 + T cells, CD4+ effectors, CD4+ effector memory T cells, CD8+ effectors, CD8+ effector memory T cells, and NK regulatory cells were significantly increased while CD4+ naïve, CD4+ central memory, CD8+ naïve, CD8+ central memory T cells, and B cells were significantly decreased at 6−8 months as compared to the baseline. However the percentages of CD4+ T cells, CD8+ T cells, NKT cells, and NK cytotoxic cells remain comparable at baseline and 6−8 months (Figure 5).

### 3.7 Analysis of T cell subsets based on age and gender in COVID‐19 patients

**Figure 6:**
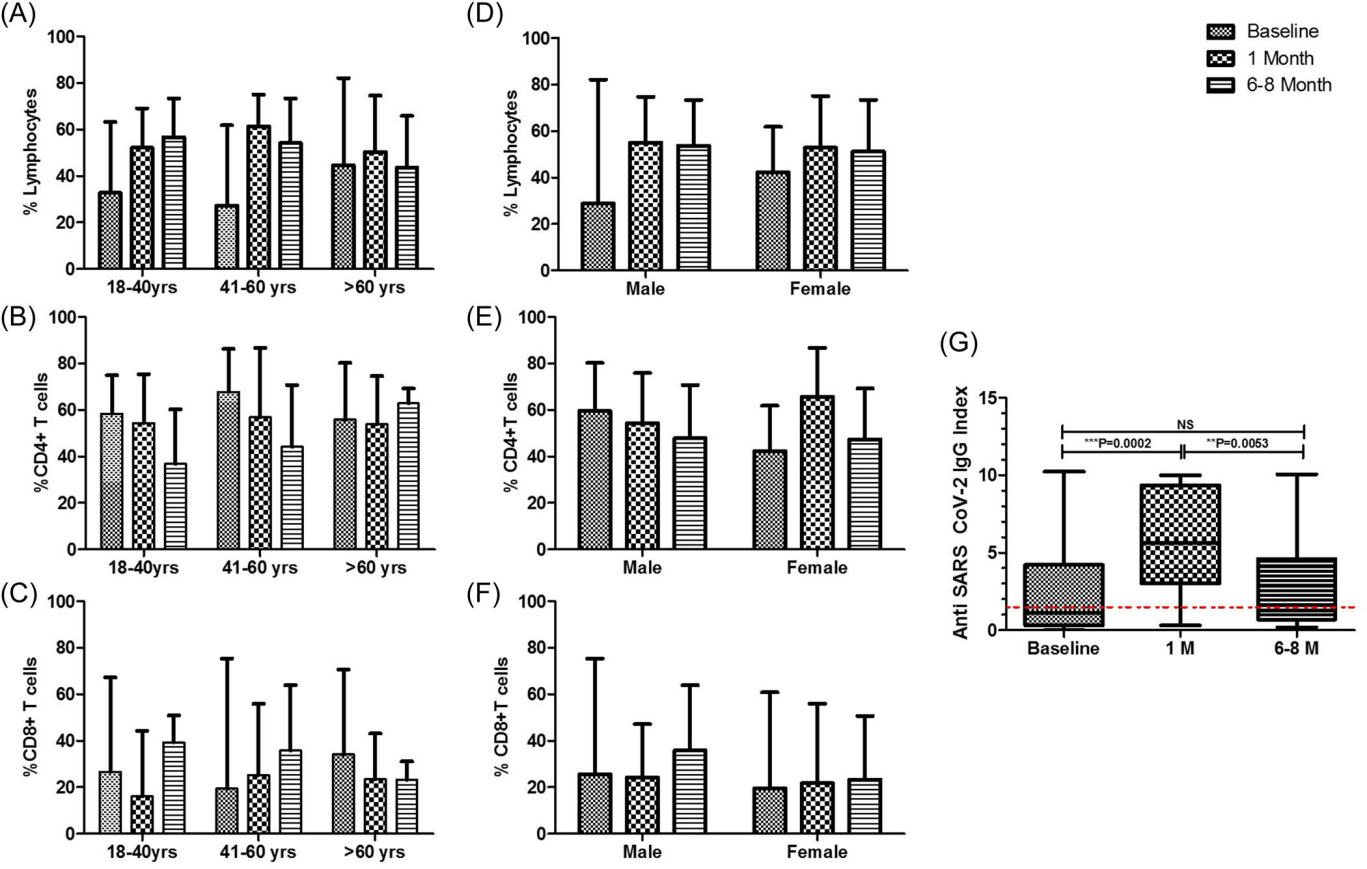
Flow cytometry analysis of T cell proportions on basis of age and gender and SARS‐CoV‐2 IgG status for COVID‐19 patients. (A−C) The bar diagram shows comparison of percentages of lymphocytes, CD4 + T cells, CD8 + T cells, respectively in COVID‐19 patients with age groups 18−40 years, 41−60 years, and above 60 years. (D−F) The bar diagram shows comparison of percentages of lymphocytes, CD4 + T cells, CD8 + T cells, respectively in COVID‐19 infected males and females. (G) The whiskers box plot shows the anti‐SARS‐CoV‐2 antibody index in COVID‐19 patients at baseline, 1 month, and after 6−8 months. The red line indicates cut off value. Data are represented as median with range. *p < .05, **p < .001, and ***p < .0001 significant.

To further investigate the impact of demographic differences on T cell proportions as the age and gender can influence the percentages of T cells in the study. In case of age we divided the patients into three groups 18−40 years, 41−60 years, and above 60 years. We found that as the age increases the percentages of lymphocytes, CD4 + T cells and CD8 + T cells decreases over a span of 6−8 months from baseline (Figure 6A−C). However in case of gender, the percentages of lymphocytes, CD4 + T cells, and CD8 + T cells remains comparable in male and females over a span of baseline to 6−8 months (Figure 6D−F) (Table 5).

**Table 5:** Comparison of T cell subsets in COVID‐19 patients on basis of age and gender.

		Baseline	1 Month	6−8 Month
(A) Comparison of T cell proportions on basis of age (years)				
Lymphocytes	18−40	32.8 (7.06−63.2)	52.1 (15.7−69.1)	56.7 (40.6−73.4)
	41−60	27.3 (9.13−61.8)	61.35 (13.8−75)	54.1 (27.8−73.4)
	>60	44.5 (13.8−82.1)	50.4 (74.5−13)	43.65 (33.4−65.9)
CD4	18−40	58.4(32−74.9)	54.4 (27.7−75.4)	36.8 (24.1−60.2)
	41−60	67.8(1.91−86.2)	56.8 (13−86.6)	44.2 (9.69−70.7)
	>60	55.8 (30−80.2)	53.8 (13.4−74.5)	62.75 (48.5−69.2)
CD8	18−40	26.6 (11.6−67.2)	16.1 (10.3−44.3)	39.35 (28.5−50.9)
	41−60	19.5 (1.96−75.4)	25.2 (2.39−55.9)	35.8 (20.7−63.9)
	>60	34.2 (8.32−70.6)	23.4 (13.2−43.1)	23.2 (10.4−31)
(B) Comparison of T cell proportions on basis of gender				
Lymphocytes	Male	28.8 (7.06−82.1)	55 (13−74.7)	53.7 (33.4−73.4)
	Female	42.2 (16.9−61.8)	52.8 (13.8−75)	51.2 (27.8−73.4)
CD4	Male	59.5 (19.5−80.2)	54.4 (13−75.9)	47.9 (9.69−70.7)
	Female	42.2 (16.9−61.8)	65.6 (27.8−86.6)	47.4 (35.8−69.2)
CD8	Male	25.55 (8.32 to −75.4)	24.7 (4.47−47.1)	35.8 (21.3−63.9)
	Female	19.5 (1.96−60.8)	21.7 (2.39−55.9)	23.2 (10.4−50.7)

### 3.8 Anti‐SARS‐CoV‐2 antibodies shows a decline over a period of 6−8 months in COVID‐19 patients

We investigated the antibody index for the SARS‐CoV‐2 in COVID‐19 patients over a span of 6−8 months. We found that the anti‐SARS‐CoV‐2 antibody index is significantly increased after 1 month (median: 5.26; range: 0.3−10) as compared to the baseline (median: 1.12; range: 0.01−10.22) which is further significantly decreased over a period of 6−8 months (median: 1.26; range: 0.19−10.06) and remain comparable to baseline (Figure 6G).

## 4 DISCUSSION

In our longitudinal study cohort, we utilized spectral flow cytometry to track T cell populations throughout the course of COVID‐19, from initial inclusion to 6−8 months postrecovery. Our findings revealed extensive modifications within the T cell compartment, notably an increase in effector and effector memory T cells persisting for 6−8 months posthospital discharge.

Lymphopenia, a common observation in COVID‐19 patients upon hospitalization,^12^, ^13^, ^14^, ^15^, ^16^ may result from T cells relocating or undergoing cell death during this stage of the disease. Notably, pyroptosis, a highly inflammatory form of cell death induced in both infected and uninfected cells, appears to contribute significantly to the intense inflammatory responses observed globally in COVID‐19 patients.^9^, ^17^, ^18^, ^19^, ^20^ Our research, along with others,' indicates that the T cell compartment is disproportionately affected compared to B or NK cells,^21^, ^22^, ^23^, ^24^ underscoring the pivotal role of T cell subsets in COVID‐19 pathogenesis. Additionally, reductions in circulating NKT cells, as demonstrated by our study and others,^25^ have been associated with severe COVID‐19 disease and unfavorable outcomes.^26^, ^27^

Memory T cells, including both general and antigen‐specific subsets, have been extensively investigated in the context of SARS‐CoV‐2 infection, although many studies have focused on shorter timeframes.^15^, ^28^, ^29^, ^30^ Long‐term studies, spanning 8−9 months, are relatively limited.^31^ Evidence from the 2003 SARS‐CoV outbreak indicates that anti‐SARS‐CoV antibodies declined to undetectable levels within 2 years,^32^ while SARS‐CoV‐specific memory T cells remained detectable even 11 years after the outbreak.^33^ Memory T cells represent a crucial and varied subset of T cells that retain antigen experience over the long term.^34^ They can be mobilized into effector cells upon reinfection or exposure.^35^ Depending on their cellular programming and phenotype, they are categorized into various central and effector memory subtypes. Among these, the effector subsets include CD45RA + CCR7− TEMRA cells, which essentially consist of effector memory T cells (TEM) that reexpress CD45RA following antigen stimulation. While the functionality of this population remains somewhat elusive, CD4 + TEMRA cells have been implicated in contributing to protective immunity.^36^

Moreover, heightened levels of virus‐specific CD8+ effector cells persist following dengue vaccination.^37^ In our study, we observed a sustained elevation of both CD4+ and CD8+ effector T cells, as well as effector memory T cells, throughout the 6−8 month duration, which contrasts with previous findings where CD4+ effector levels remained unaffected by COVID‐19.^38^ Previous research has demonstrated an increase in the CD8 + TEMRA population upon hospitalization,^39^, ^40^ maintained for up to 6 weeks.^39^ The precise role of CD8+ effectors in COVID‐19 remains largely uncertain, although Cohen et al.^31^ noted an increase in SARS‐CoV‐2‐specific CD8+ effector cells over time. In our investigation, we examined the entire expanded CD8+ effector and CD8+ effector memory T cell population, without confirming if there was a larger fraction of antigen‐exposed, that is, SARS‐CoV‐2‐specific, T cells within the cohort.

Our research revealed that all COVID‐19 patients exhibited an increase in effector T cells and effector memory T cells, which continued to rise over time. These findings align with other studies demonstrating that SARS‐CoV‐2 infection leads to heightened expansion of antigen‐specific CD4+ and CD8 + T cell subsets.^41^, ^42^ However, it remains uncertain whether the lower antigen‐specific responses observed at 1 month compared to 6−8 months are attributable to overall immunosuppression^43^ or a natural progression of the immune response over time.^44^

Currently, all immunocompetent individuals develop SARS‐CoV‐2‐specific antibodies, a trend evident in our study as well. While some research indicates that these antibodies are detectable for only a few months postinfection,^45^, ^46^ others suggest their persistence for at least 6 months.^47^, ^48^ In our cohort, we observed that the increase in B cells endured for 6−8 months postinfection, despite experiencing a significant decline at some point after 6 weeks. Our findings align with those of Björkander et al.,^42^ who reported that antibody responses lasted up to 8 months in young adults. Even as antibody levels may decline, as observed in our study, affinity maturation continues, potentially enhancing the potency of SARS‐CoV‐2‐specific humoral immunity to provide protection against severe disease and increase the ability to neutralize the virus.^49^

In our study cohort, only 2−3 COVID‐19 patients showed post‐COVID‐19 symptoms like fatigue, dyspnea, and limitation of activities which is often referred as long COVID or postacute sequelae of SARS‐CoV‐2 infection (PASC), can impact immune responses in several ways.^50^, ^51^, ^52^ Persistent symptoms such as fatigue, cognitive impairment, and respiratory issues may reflect ongoing immune dysregulation or inflammation. Chronic activation of the immune system could lead to exhaustion of immune cells, impaired functionality, or autoimmunity. Additionally, the presence of viral reservoirs or lingering viral particles may sustain immune activation, contributing to prolonged inflammation and tissue damage. Understanding these interactions between long‐term symptoms and immune responses is crucial for developing effective therapies and interventions for individuals with persistent COVID‐19 symptoms.

Our study focused on moderate and severe COVDI‐19 patients, the observed immune responses likely have relevance to milder or asymptomatic cases as well. The findings suggest that even in milder or asymptomatic cases of COVID‐19, T cell responses may play a crucial role in providing long‐term immunity. This indicates that the immune system mounts a robust response to the virus, potentially conferring lasting protection against reinfection. Further research into the immune dynamics of milder cases is warranted to fully understand the implications for long‐term immunity and disease management.

Our study examined various immune cells and their subsets in COVID‐19 patients, yet we did not conduct an in‐depth analysis of regulatory T cells. This represents one of the limitation of our research.

Despite the formidable challenges posed by the pandemic, we believe that the cohort examined in this investigation is well characterized and offers significant value. This study sheds light on the shifts in immune response seen during hospital‐treated SARS‐CoV‐2 infection and recovery. Our longitudinal data reveal profound alterations in the T cell landscape persisting for over 6−8 months. These findings, alongside others, contribute crucial insights into SARS‐CoV‐2 cellular and humoral immunity, paving the way for further exploration and enhanced comprehension of the long‐term immunopathogenic changes observed in COVID‐19.

## AUTHOR CONTRIBUTIONS

Poonam Suryawanshi contributed to execution of the study, performed the flow cytometry experiments, data collection, analysis of data, interpretation of results, and writing the manuscript. Bhagyashri Patil‐Takbhate contributed to the study methodology and data collection. Prachi Athavale, Shubhangi Kanitkar, Sachin Shivnitwar, Madhusudan S. Barthwal, Sachin Dole, Hanumant Chavan, Priyanka Jali, Sujata Pawale, and Dhanashree Kakad contributed to the enrollment of study participants in the study. Shahzad Mirza involved in COVID‐19 testing of the enrolled study samples. Anuradha Tripathy design and Conduct of the immunological assays for the study. Madhura Gandhi contributed to statistical data analysis of manuscript. Arjun Lal Kakrani, Jitendra Bhawalkar, Sarika Chaturvedi, Mahesh Karandikar, and Srikanth Tripathy contributed to conceptualize the study, study coordination, and data collection. Srikanth Tripathy contributed to conceptualization of study, writing of the project proposal, obtaining regulatory approvals, and funding for carrying out the study, study design, execution of the study, data curation, review, and finalization of manuscript. All authors have read and approved the final manuscript.

## CONFLICT OF INTEREST STATEMENT

The authors declare no conflict of interest.

## ETHICS STATEMENT

The studies involving human participants were reviewed and approved by Institutional Ethics Review Board of Dr D. Y. Patil Vidyapeeth, (deemed to be University) Pune. The patients/participants provided their written informed consent to participate in this study.

## Supporting information

Supporting information.

Supporting information.

## Data Availability

The data that support the findings of this study are available on request from the corresponding author. The data are not publicly available due to privacy or ethical restrictions.

## References

[1] Osuchowski MF , Winkler MS , Skirecki T , et al. The COVID‐19 puzzle deciphering pathophysiology and phenotypes of a new disease entity. Lancet Resp Med. 2021;9(6):622‐642. 10.1016/S2213-2600(21)00218-6 PMC8102044 33965003

[2] Venet F , Cour M , Rimmelé T , et al. Longitudinal assessment of IFN‐I activity and immune profile in critically ill COVID‐19 patients with acute respiratory distress syndrome. Crit Care. 2021;25(1):140. 33845874 10.1186/s13054-021-03558-w PMC8040759

[3] Remy KE , Mazer M , Striker DA , et al. Severe immunosuppression and not a cytokine storm characterizes COVID‐19 infections. JCI Insight. 2020;5(17):e140329. 32687484 10.1172/jci.insight.140329 PMC7526441

[4] Interim guidance. (n.d.) . Diagnostic testing for SARS‐CoV. Who.int. Accessed February 1, 2024. https://iris.who.int/bitstream/handle/10665/334254/WHO-2019-nCoV-laboratory-2020.6-eng.pdf?sequence=1

[5] NIH . COVID‐19 treatment guidelines panel. Coronavirus disease 2019 (COVID‐19) treatment guidelines. 2020. https://www.covid19treatmentguidelines.nih.gov/

[6] Ni L , Ye F , Cheng ML , et al. Detection of SARSCoV‐2‐Specific humoral and cellular immunity in COVID‐19 convalescent individuals. Immunity. 2020;52(6):971‐977. 10.1016/j.immuni.2020.04.023 32413330 PMC7196424

[7] Ghizlane EA , Manal M , Abderrahim EK , et al. Lymphopenia in covid‐19: a single center retrospective study of 589 cases. Ann Med Surg. 2021;69:102816. 10.1016/j.amsu.2021.102816 PMC8423773 34512964

[8] Lee J , Park SS , Kim TY , Lee DG , Kim DW . Lymphopenia as a biological predictor of outcomes in COVID‐19 patients: a nationwide cohort study. Cancers. 2021;13(3):471. 10.3390/cancers13030471 33530509 PMC7865511

[9] Ramljak D , Vukoja M , Curlin M , et al. Early response of CD8+ T cells in COVID‐19 patients. J Pers Med. 2021;11(12):1291. 10.3390/jpm11121291 34945761 PMC8704882

[10] He R , Lu Z , Zhang L , et al. The clinical course and its correlated immune status in COVID‐19 pneumonia. J Clin Virol. 2020;127:104361. 10.1016/j.jcv.2020.104361 32344320 PMC7152870

[11] Chen Z , John Wherry E . T cell responses in patients with COVID‐19. Nat Rev Immunol. 2020;20(9):529‐536. 10.1038/s41577-020-0402-6 32728222 PMC7389156

[12] Suryawanshi P , Takbhate B , Athavale P , et al. Lymphopenia with altered T cell subsets in hospitalized COVID‐19 patients in Pune, India. Viral Immunol. 2023;36(3):163‐175. 10.1089/vim.2022.0123 36897333

[13] Fathi N , Rezaei N . Lymphopenia in COVID‐19: therapeutic opportunities. Cell Biol Int. 2020;44(9):1792‐1797. 10.1002/cbin.11403 32458561 PMC7283672

[14] Schultheiß C , Paschold L , Simnica D , et al. Next‐generation sequencing of T and b cell receptor repertoires from COVID‐19 patients showed signatures associated with severity of disease. Immunity. 2020;53(2):442‐455.e4. 10.1016/j.immuni.2020.06.024 32668194 PMC7324317

[15] Sekine T , Perez‐Potti A , Rivera‐Ballesteros O , et al. Robust T cell immunity in convalescent individuals with asymptomatic or mild COVID‐19. Cell. 2020;183(1):158‐168. 10.1016/j.cell.2020.08.017 32979941 PMC7427556

[16] Wilk AJ , Rustagi A , Zhao NQ , et al. A single‐cell Atlas of the peripheral immune response in patients with severe COVID‐19. Nature Med. 2020;26(7):1070‐1076. 10.1038/s41591-020-0944-y 32514174 PMC7382903

[17] Li S , Zhang Y , Guan Z , et al. SARS‐CoV‐2 triggers inflammatory responses and cell death through caspase‐8 activation. Signal Transduct Target Ther. 2020;5(1):235. 10.1038/s41392-020-00334-0 33037188 PMC7545816

[18] Ponti G , Maccaferri M , Ruini C , Tomasi A , Ozben T . Biomarkers associated with COVID‐19 disease progression. Crit Rev Clin Lab Sci. 2020;57(6):389‐399. 10.1080/10408363.2020.1770685 32503382 PMC7284147

[19] Chen G , Wu D , Guo W , et al. Clinical and immunological features of severe and moderate coronavirus disease 2019. J Clin Invest. 2020;130(5):2620‐2629. 10.1172/JCI137244 32217835 PMC7190990

[20] Guan W , Ni Z , Hu Y , et al. Clinical characteristics of coronavirus disease 2019 in China. N Engl J Med. 2020;382(18):1708‐1720. 10.1056/NEJMoa2002032 32109013 PMC7092819

[21] Zhang S , Asquith B , Szydlo R , Tregoning JS , Pollock KM . Peripheral T cell lymphopenia in COVID‐19: potential mechanisms and impact. Immunotherapy Advances. 2021;1(1):ltab015. 10.1093/immadv/ltab015 35965490 PMC9364037

[22] Bao J , Li C , Zhang K , Kang H , Chen W , Gu B . Comparative analysis of laboratory indexes of severe and non‐severe patients infected with COVID‐19. Clin Chim Acta. 2020;509:180‐194. 10.1016/j.cca.2020.06.009 32511971 PMC7274996

[23] Qin C , Zhou L , Hu Z , et al. Dysregulation of immune response in patients with coronavirus 2019 (COVID‐19) in wuhan, China. Clin Infect Dis. 2020;71(15):762‐768. 10.1093/cid/ciaa248 32161940 PMC7108125

[24] Kalfaoglu B , Almeida‐Santos J , Tye CA , Satou Y , Ono M . T‐Cell hyperactivation and paralysis in severe COVID‐19 infection revealed by singlecell analysis. Front Immunol. 2020;11:589380. 10.3389/fimmu.2020.589380 33178221 PMC7596772

[25] Zingaropoli MA , Perri V , Pasculli P , et al. Major reduction of NKT cells in patients with severe COVID‐19 pneumonia. Clin Immunol. 2021;222:108630. 10.1016/j.clim.2020.108630 33189887 PMC7661928

[26] Kreutmair S , Unger S , Núñez NG , et al. Distinct immunological signatures discriminate severe COVID‐19 from non‐SARS‐CoV‐2‐driven critical pneumonia. Immunity. 2021;54(7):1578‐1593.e5. 10.1016/j.immuni.2022.01.015 34051147 PMC8106882

[27] Jouan Y , Guillon A , Gonzalez L , et al. Phenotypical and functional alteration of unconventional T cells in severe COVID‐19 patients. J Exp Med. 2020;217(12):e20200872. 32886755 10.1084/jem.20200872 PMC7472174

[28] Gong F , Dai Y , Zheng T , et al. Peripheral CD4+ T cell subsets and antibody response in COVID‐19 convalescent individuals. J Clin Invest. 2020;130(12):6588‐6599. 10.1172/JCI141054 32841212 PMC7685722

[29] Kundu R , Narean JS , Wang L , et al. Crossreactive memory T cells associate with protection against SARS‐CoV‐2 infection in COVID‐19 contacts. Nat Commun. 2022;13(1):80. 10.1038/s41467-021-27674-x 35013199 PMC8748880

[30] Kared H , Redd AD , Bloch EM , et al. SARSCoV‐2‐specific CD8+ T cell responses in convalescent COVID‐19 individuals. J Clin Invest. 2021;131(5):e145476:e145476. 10.1172/JCI145476 33427749 PMC7919723

[31] Cohen KW , Linderman SL , Moodie Z , et al. Longitudinal analysis shows durable and broad immune memory after SARS‐CoV‐2 infection with persisting antibody responses and memory B and T cells. Cell Rep Med. 2021;2(7):100354. 10.1016/j.xcrm.2021.10035 34250512 PMC8253687

[32] Cao W‐C , Liu W , Zhang P‐H , Zhang F , Richardus JH . Disappearance of antibodies to SARS‐associated coronavirus after recovery. N Engl J Med. 2007;357(11):1162‐1163. 10.1056/NEJMc070348 17855683

[33] Ng O‐W , Chia A , Tan AT , et al. Memory T cell responses targeting the SARS coronavirus persist up to 11 years post‐infection. Vaccine. 2016;34(17):2008‐2014. 10.1016/j.vaccine.2016.02.063 26954467 PMC7115611

[34] MacLeod MKL , Kappler JW , Marrack P . Memory CD4 T cells: generation, reactivation and re‐assignment. Immunology. 2010;130(1):10‐15. 10.1111/j.1365-2567.2010.03260.x 20331469 PMC2855788

[35] Farber DL , Yudanin NA , Restifo NP . Human memory T cells: generation, compartmentalization and homeostasis. Nat Rev Immunol. 2014;14(1):24‐35. 10.1038/nri3567 24336101 PMC4032067

[36] Tian Y , Babor M , Lane J , et al. Unique phenotypes and clonal expansions of human CD4 effector memory T cells re‐expressing CD45RA. Nat Commun. 2017;8(1):1473. 10.1038/s41467-017-01728-5 29133794 PMC5684192

[37] Graham N , Eisenhauer P , Diehl SA , et al. Rapid induction and maintenance of virus‐specific CD8(+) T(EMRA) and CD4(+) T(EM) cells following protective vaccination against dengue virus challenge in humans. Front Immunol. 2020;11:479. 10.3389/fimmu.2020.00479 32265929 PMC7105617

[38] Govender M , Hopkins FR , Göransson R , et al. T cell perturbations persist for at least 6 months following hospitalization for COVID‐19. Front Immunol. 2022;13:931039. 10.3389/fimmu.2022.931039 36003367 PMC9393525

[39] Bonifacius A , Tischer‐Zimmermann S , Dragon AC , et al. COVID‐19 immune signatures reveal stable antiviral T cell function despite declining humoral responses. Immunity. 2021;54(2):340‐354. 10.1016/j.immuni.2021.01.008 33567252 PMC7871825

[40] Zenarruzabeitia O , Astarloa‐Pando G , Terrén I , et al. T cell activation, highly armed cytotoxic cells and a shift in monocytes CD300 receptors expression is characteristic of patients with severe COVID‐19. Front Immunol. 2021;12:655934. 10.3389/fimmu.2021.655934 33777054 PMC7991729

[41] Le Bert N , Tan AT , Kunasegaran K , et al. SARS‐CoV‐2‐specific t cell immunity in cases of Covid‐19 and SARS, and uninfected controls. Nature. 2020;584(7821):457‐462. 10.1038/s41586-020-2550-z 32668444

[42] Björkander S , Du L , Zuo F , et al. SARS‐CoV‐2‐specific B‐ and T‐cell immunity in a population‐based study of young Swedish adults. J Allergy Clin Immunol. 2022;149(1):65‐75. 10.1016/j.jaci.2021.10.014 34695490 PMC8536496

[43] Kalfaoglu B , Almeida‐Santos J , Tye CA , Satou Y , Ono M . T‐Cell dysregulation in COVID‐19. Biochem Biophys Res Commun. 2021;538:204‐210. 10.1016/j.bbrc.2020.10.079 33220925 PMC7648511

[44] Zuo J , Dowell AC , Pearce H , et al. Robust SARSCoV‐2‐specific T cell immunity is maintained at 6 months following primary infection. Nature Immunol. 2021;22(5):620‐626. 10.1038/s41590-021-00902-8 33674800 PMC7610739

[45] Long QX , Tang XJ , Shi QL , et al. Clinical and immunological assessment of asymptomatic SARS‐CoV‐2 infections. Nature Med. 2020;26(8):1200‐1204. 10.1038/s41591-020-0965-6 32555424

[46] Ibarrondo FJ , Fulcher JA , Goodman‐Meza D , et al. Rapid decay of anti‐SARS‐CoV‐2 antibodies in persons with mild covid‐19. N Engl J Med. 2020;383(11):1085‐1087. 10.1056/NEJMc2025179 32706954 PMC7397184

[47] Sherina N , Piralla A , Du L , et al. Persistence of SARS‐CoV‐2‐specific B and T cell responses in convalescent COVID‐19 patients 6–8 months after the infection. Med. 2021;2(3):281‐295. 10.1016/j.medj.2021.02.001 33589885 PMC7874960

[48] Dan JM , Mateus J , Kato Y , et al. Immunological memory to SARS‐CoV‐2 assessed for up to 8 months after infection. Science. 2021;371(6529):eabf4063. 10.1126/science.abf4063 33408181 PMC7919858

[49] Muecksch F , Weisblum Y , Barnes CO , et al. Affinity maturation of SARS‐CoV‐2 neutralizing antibodies confers potency, breadth, and resilience to viral escape mutations. Immunity. 2021;54(8):1853‐1868. 10.1016/j.immuni.2021.07.008 34331873 PMC8323339

[50] Carfì A , Bernabei R , Landi F . Persistent symptoms in patients after acute COVID‐19. JAMA. 2020;324(6):603‐605. 10.1001/jama.2020.12603 32644129 PMC7349096

[51] Huang C , Huang L , Wang Y , et al. RETRACTED: 6‐month consequences of COVID‐19 in patients discharged from hospital: a cohort study. Lancet. 2021;397(10270):220‐232. 10.1016/S0140-6736(20)32656-8 33428867 PMC7833295

[52] Xie Y , Xu E , Bowe B , Al‐Aly Z . Long‐term cardiovascular outcomes of COVID‐19. Nature Med. 2022;28:583‐590. 10.1038/s41591-022-01689-3 35132265 PMC8938267

