# Supplementary material for: T‐cell responses in COVID‐19 survivors 6−8 months after infection: A longitudinal cohort study in Pune
Source: Immun Inflamm Dis. 2024 Jun 11;12(6):e1238. doi: 10.1002/iid3.1238 (PMC11165687; doi:10.1002/iid3.1238)

**Supplementary Material 1-Gatening strategy for different immune cells and subsets using flow cytometry.**

Gating strategy to identify the predominant T-cell subsets and T-cell subpopulations is depicted in Figure 1. Lymphocytes were identified on the basis of forward and side scatter (Fig 1A) and CD3+ve cells were gated on basis of presence of the CD3 marker (Fig 1B). Subsequently the CD3+CD4+T cells and the CD3+CD8+ T cells (Fig 1C) were identified. Gating on CD3+CD8+T cells, CD45RA negative and CD45RA positive CD8+ T cells were identified (Fig 1D). Gating on CD8+CD45RA+, in (Fig 1E), the expression of CCR-7 and marker CD62L was used to define CD8+ Naïve T cells (CD8+CD45RA+CCR7+CD62L+) and CD8+ Effector T cells (CD8+CD45RA+CCR7-CD62L-). Gating on CD8+CD45RA-, in (Fig 1F), the expression of CCR-7 and CD62L was used to define CD8+ Central memory T (TCM) cells (CD8+CD45RA-CCR7+CD62L+) and CD8+ Effector memory T (TEM) cells(CD8+CD45RA-CCR7-CD62L-). Gating on CD3+CD4+T cells, CD45RA negative and CD45RA positive CD4+ T cells were identified (Fig 1G). Gating on CD4+CD45RA+, in (Fig 1H), the expression of the chemokine receptor CCR-7 and marker CD62L was used to define CD4+ Naïve T (TN) cells (CD4+CD45RA+CCR7+CD62L+) and CD4+ Effector T (TEf) cells (CD4+CD45RA+CCR7-CD62L-) . Gating on CD4+CD45RA-, in (Fig 1I), the expression of CCR-7 and CD62L was used to define CD4+ Central memory T (TCM) cells (CD4+CD45RA-CCR7+CD62L+) and CD4+ Effector memory T (TEM) cells(CD4+CD45RA-CCR7-CD62L).


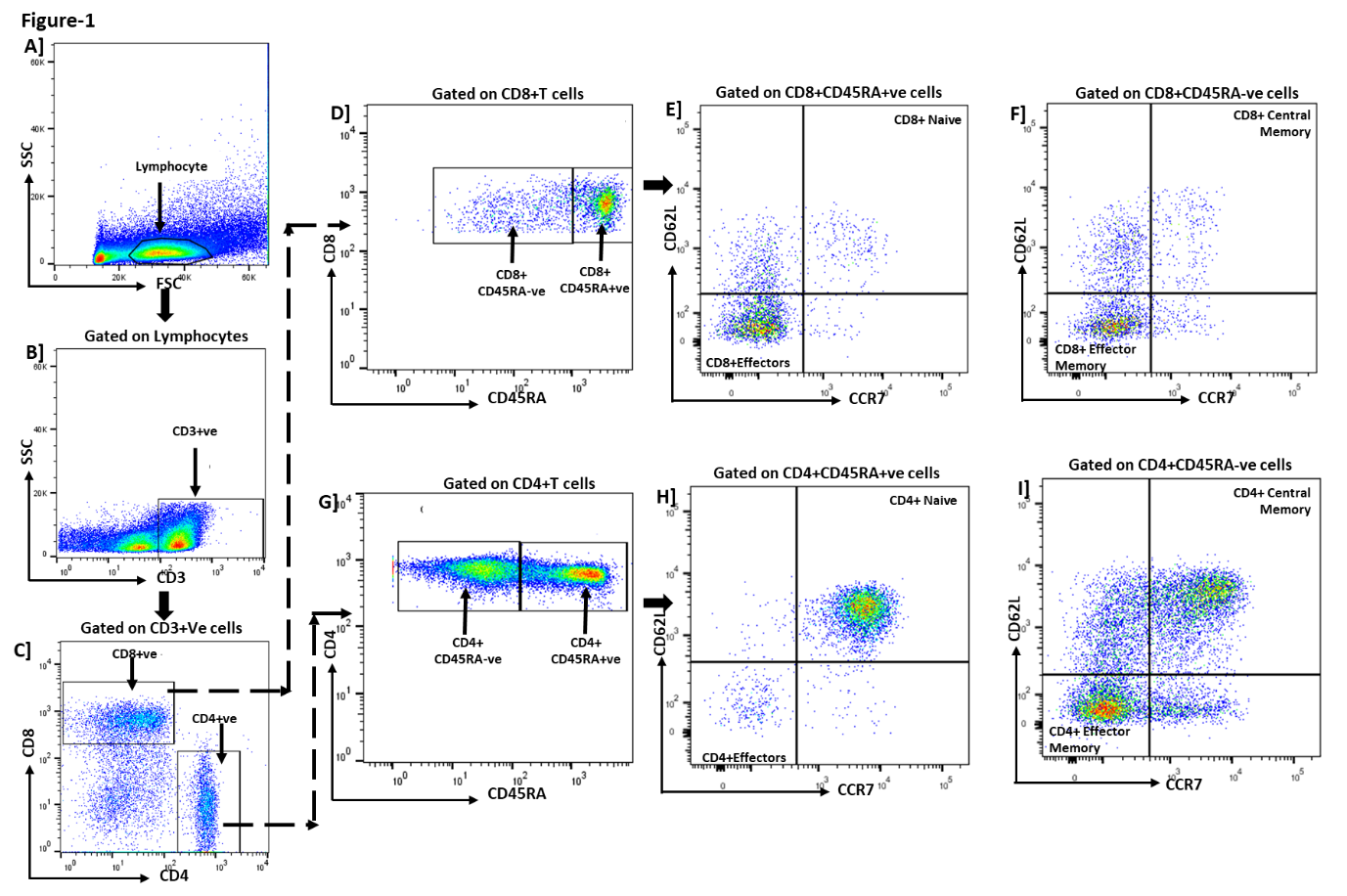


The gating strategy for NKT cells, NK cells and B cells is represented in Figure 2. Lymphocytes were identified based on forward and side scatter (Fig 2A) and CD3+ve/CD3-ve cells were gated on basis of the presence/absence of marker respectively [Fig 2B]. Subsequently the CD3+CD56+ cells were identified as NKT population (Fig 2C). Further gating on CD3-ve cells ,CD16 vs. CD56 ,NK cytotoxic cells (CD16+CD56+) cells and NK regulatory (CD16-CD56+) cells were identified (Fig 2D) while CD3-CD19+ cells were identified as B cells (Fig 2E).


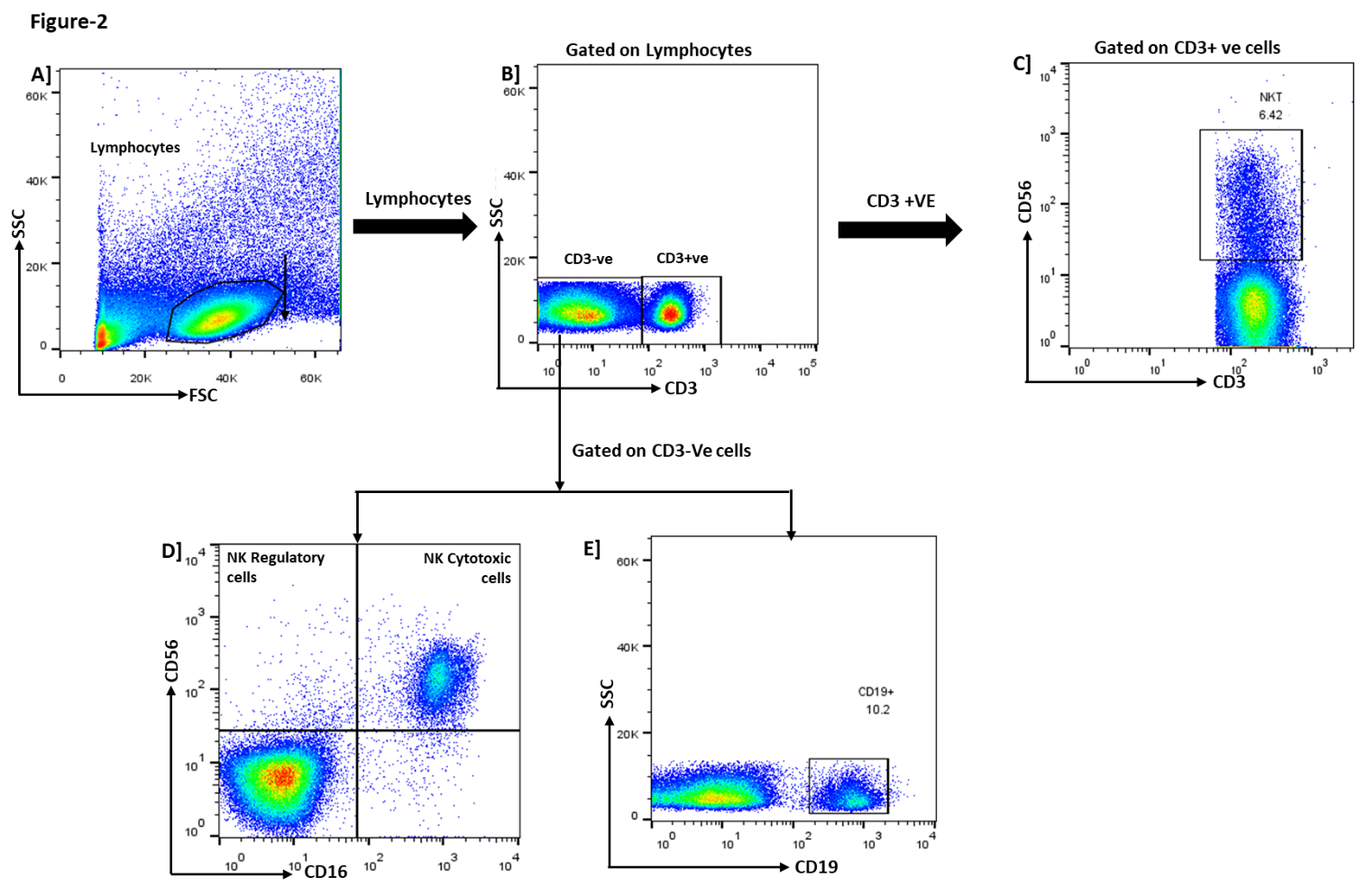

Supplement: Supplementary file 2 — Supporting information. [file IID3-12-e1238-s001.docx]
